# Anywhere anytime: opportunities and limitations of digital psychotherapies

**DOI:** 10.1192/j.eurpsy.2026.10290

**Published:** 2026-07-31

**Authors:** I. G. Yilmaz-Karaman, EPA Psychotherapy Working Group

**Affiliations:** Psychiatry, Eskişehir Osmangazi University, Eskişehir, Türkiye

## Abstract

**Abstract:**

Evidence indicates that therapist-guided digital interventions, particularly internet-delivered cognitive behavioral therapy and videoconferencing-based psychotherapy, can achieve clinical outcomes comparable to in-person treatment for common mental disorders such as depression and anxiety when applied to appropriately selected patients. Key clinical opportunities include improved access to care, flexibility in treatment delivery, support for stepped and blended care models, and enhanced symptom monitoring between sessions. Emerging technologies such as virtual reality and just-in-time adaptive interventions may further augment established therapeutic techniques. Besides, digital psychotherapies have the potential to narrow the treatment gap during disasters.

At the same time, clinically relevant limitations must be acknowledged. Engagement and adherence remain inconsistent, especially in unguided interventions, with high dropout rates reported in routine care. Digital formats may limit clinicians’ ability to assess acute risk, comorbidity, and subtle clinical cues, and many digital interventions exclude patients with high clinical complexity. Additional concerns include data privacy, variable evidence quality, unclear liability, and insufficient regulatory oversight. For those with limited internet access and a lack of privacy, digital psychotherapies become unsustainable. That causes a gap between practice and theory.

Digital psychotherapies should therefore be understood as complementary tools rather than replacements for face-to-face care. Their effective clinical use requires careful patient selection, ongoing human oversight, and integration within well-governed mental health care systems.

**Disclosure of Interest:**

None Declared

